# Bridging the gaps in the Health Management Information System in the context of a changing health sector

**DOI:** 10.1186/1472-6947-10-36

**Published:** 2010-06-25

**Authors:** Angelo S Nyamtema

**Affiliations:** 1 Tanzanian Training Centre for International Health, Ifakara, Tanzania

## Abstract

**Background:**

The Health Management Information System (HMIS) is crucial for evidence-based policy-making, informed decision-making during planning, implementation and evaluation of health programs; and for appropriate use of resources at all levels of the health system. This study explored the gaps and factors influencing HMIS in the context of a changing health sector in Tanzania.

**Methods:**

A cross sectional descriptive study was conducted in 11 heath facilities in Kilombero district between January and February 2008. A semi-structured questionnaire was used to interview 43 health workers on their knowledge, attitude, practice and factors for change on HMIS and HMIS booklets from these facilities were reviewed for completeness.

**Results:**

Of all respondents, 81% had never been trained on HMIS, 65% did not properly define this system, 54% didn't know who is supposed to use the information collected and 42% did not use the collected data for planning, budgeting and evaluation of services provision. Although the attitude towards the system was positive among 91%, the reviewed HMIS booklets were never completed in 25% - 55% of the facilities. There were no significant differences in knowledge, attitude and practice on HMIS between clinicians and nurses. The most common type of HMIS booklets which were never filled were those for deliveries (55%). The gaps in the current HMIS were linked to lack of training, inactive supervision, staff workload pressure and the lengthy and laborious nature of the system.

**Conclusions:**

This research has revealed a state of poor health data collection, lack of informed decision-making at the facility level and the factors for change in the country's HMIS. It suggests need for new innovations including incorporation of HMIS in the ongoing reviews of the curricula for all cadres of health care providers, development of more user-friendly system and use of evidence-based John Kotter's eight-step process for implementing successful changes in this system.

## Background

A health management information system (HMIS) is a process whereby health data are recorded, stored and processed for policy-making, planning, implementation and evaluation of health programs. The system is crucial for evidence-based policy and informed decision-making at all levels from national down to the institutional levels. Evidence-based decision making is critically important for the appropriate use of scarce resources particularly in resource limited countries like Tanzania. The HMIS in most developing countries are inefficient and are greatly affected by unreliability of data resulting from underreporting [1]. Reports from sub Saharan Africa indicate that vital health decisions, in this context, are made based on crude estimates of disease and treatment burdens [2,3]. Findings from this region indicate that the problem of under reporting is huge and is linked to lack of knowledge and practice among the health workers characterized by insufficient analysis skills, training and lack of initiative for using information [4-6].

In Tanzania the first version of the health management information system was launched in 1993 and the second in 1998 [7]. The first version was entirely in English and it was soon realized upon testing that the users had limited commands in this language and was therefore technically changed to Kiswahili, the national language. Thus, the Health Management Information System is called in Kiswahili as *Mfumo wa Taarifa za Huduma za Afya *(*MTUHA*). The latest version involves manual data entry into 12 HMIS booklets. The system covers all health programs and health care services, and requires all health facilities, regardless of ownership, to use this system and report to the district health authority on quarterly basis. The overall goal of this system is to optimize the performance of health services at all levels of administration through the timely provision of necessary and sufficient information needed by the health managers to monitor, evaluate and plan their activities [7,8]. Its success requires a system that is integrated, decentralized, functional and reliable [9].

The conception of this study was based on the concerns about the poor quality data and inadequate integration of the HMIS despite a number of changes it has undergone since inception and the need to bridge the gaps in the on-going changes in health sector. In an attempt to strengthen the health services to meet national and international commitments, the government of Tanzania has developed the Primary Health Service Development Program (PHSDP) whose main goal is to accelerate provision of quality primary health care services to all by 2017. This program is implemented by increasing intakes of trainees for health care, reviewing and standardizing curricula for all medical and paramedical cadres to competence-based [10]. The review of the curricula is spearheaded by the National Council for Technical Education (NACTE) an organ which was empowered by the government through the Act No. 9 of 1997 to coordinate technical education and set qualification standards for the awards. This article explores the gaps and factors for change in HMIS in Tanzania and presents a detailed account on how they could be best bridged in the ongoing changes in country's health sector. It attempts to link the required changes in the HMIS and the evidence-based John Kotter's eight-step process for implementing successful changes in any organizations [11].

## Methods

### Study area and design

Tanzania is divided into 25 administrative regions that are subdivided into 113 districts that are further subdivided into divisions, wards and villages. Administratively, the ministry of health is the main coordinating body for health information in the country, the regional level is responsible for coordinating activities in the districts and the districts are responsible for coordinating services delivery activities at the health facility levels. The 12 HMIS booklets in Tanzania include the guidelines, summary (from the other books), village profile, inventory (ledger for equipment, drugs and supplies), outpatient services, antenatal services, postnatal services, family planning services, communicable diseases, HMIS report book, dental services and delivery services. These booklets consist of forms and registers, where the registers are pre-set frameworks for data processing.

In an attempt to map the gaps and factors for change in the country's HMIS a cross-sectional descriptive study was conducted between January and February 2008 in Kilombero, one of the most rural districts in Tanzania. Kilombero district is in the southeastern part of the country about 230 km from Morogoro, the headquarters of the region and 420 km from Dar es Salaam, the largest business city in the Tanzania. The district has a total area of 14,018 km ^2^, a population of 321,611 people [12] and 44 health facilities. Among these health facilities are 2 hospitals both owned by non-governmental institutions, 4 health centres (all owned by the government) and 38 dispensaries of which only 15 are owned by the government. The health facilities were as far as 180 km from the district headquarters and are expected to provide all primary health care services, refer complicated cases and complete the relevant booklets.

### Sampling and size

A stratified random sampling technique was used to obtain one hospital, one health centre and 9 dispensaries. Of these facilities, 5 were governmental and 6 non-governmental. A total of 11 health facilities were involved in the analysis, fulfilling WHO recommendation to cover at least 25-30% of the health facilities in the area when assessing quality of care [13,14]. The study team aimed to interview at least 5 health care providers including those in-charge of the health facility available on the day of study visit. However, the team managed to interview 43 care providers because many of the facilities had less than five health care providers. At the hospital a list of care providers was obtained from the administration and the following departments were included: outpatient, reproductive and child health (RCH) clinic and labour ward where at least 2 health workers were interviewed from each department.

### Data collection, processing and analysis

Data collection was carried out by the author and 4 research assistants. A semi-structured questionnaire was used to interview health care providers and facility administrators to assess their level of knowledge, attitudes and practices concerning HMIS and factors for change (Additional file 1). After the interview the research team requested to see the 2007 HMIS booklets (number 6, 7 & 12) in order to review the completeness of records. The parameters recorded in booklet 6 (antenatal services register) included: 1) booking visit: date, registration number, name, age, gravidity, gestation age, height, danger signs; 2) re-attendance visits: presence of anaemia, oedema, proteinuria, lie of the foetus, vaginal bleeding, syphilis test, date of TT vaccine for the index pregnancy, last childbirth (year, live or died) and referral information. The parameters for book 7 (underfive services register) were: date, registration number, date of birth, weight, date for BCG, DPT, polio, measles vaccinations and vitamin A, mother's information (name and TT vaccination status); and those for book 12 (delivery services register) were: date, registration number, name of the mother, age, gravidity, parity, date of admission, date of delivery, mode of delivery, birth before arrival (BBA), complication of labour, status at birth (live birth or stillbirth), condition of the mother at discharge and name of the health provider. The parameters which were mostly not recorded were documented. In the cases of frequent incomplete records, the research team inquired about reasons for the incompleteness. The research team also interviewed the district HMIS coordinator for the factors that affected health information system. The data were entered in the Statistical Package for Social Science (SPSS) version 10 and analyzed by generating frequencies. Exact binomial confidence intervals at 95% were used to compare the proportions of clinicians and nurses with regards to the training, knowledge, attitude and utilization of HMIS data in their health facilities. The permission to carry out this study was obtained from district medical authority and verbal consent was obtained from the interviewees.

## Results

### Characteristics of respondents

A total of 43 health care providers from 11 health facilities were interviewed. Among these 23 (53%) were clinicians and 20 (47%) nurses. While the clinicians included 20 (47%) clinical officers and medical officers 3 (7%), the nurses included 8 (19%) nurse officers, 11 (26%) enrolled nurses and 1 (2%) medical attendant.

### Training and knowledge on HMIS

More than three quarters (81%) of respondents had never been trained on HMIS (Table 1). There was no statistically significant difference in proportions of health workers trained for HMIS between the clinicians 17% (95% CI: 2% - 32%) and nurses 20% (95% CI: 2% - 38%). Almost two thirds (65%) failed to define properly what HMIS is. Of the respondents, only 7% recalled 7-9 booklets, 18% recalled 5-6 booklets, 42% mentioned 1-5 booklets and more than one third (33%) failed to recall even one out of twelve HMIS booklets. While 54% did know who are supposed to use the information collected at the health facility, 40% didn't know the importance of HMIS. There was no significant difference in knowledge about the importance of HMIS between clinicians 61% (95% CI: 41% - 81%) and nurses 60% (95% CI: 41% - 81%). On the other hand, more than one third (37%) of all respondents did not know the HMIS information flow pattern.

**Table 1:** Proportions of health workers with training, knowledge, positive attitude and utilized HMIS data in Kilombero district

	Clinicians n = 23		Nurses n = 20		Total n = 43	P value
		
	%	95% CI	%	95% CI	%	
Trained on HMIS	17%	2% - 32%	20%	2% - 38%	19%	0.826
Defined properly HMIS	17%	2% - 32%	55%	33% - 77%	35%	0.010
Knew the importance of HMIS	61%	41% - 81%	60%	41% - 81%	60%	0.954
Positive attitude towards HMIS	87%	74% - 99%	95%	93% - 98%	91%	0.365
Utilized HMIS data for decision making	52%	32% - 72%	65%	44% - 86%	58%	0.395

Note: CI = Confidence interval

### Attitude towards HMIS

Generally almost all respondents (91%) had positive attitude towards HMIS. There was no significant difference in attitudes of health workers towards HMIS between clinicians 87% (95% CI: 74% - 99%) and nurses 95% (95% CI: 93% - 98%). Thirty nine respondents (91%) agreed that the system (HMIS) was worthy for the time and other resources spent filling and processing data, and that it was important to continue with the system. However, 42% of the respondents pointed out that the current HMIS was difficult, complicated and that it needed to be simplified. Although they were generally positive they needed a better system.

### Practice on HMIS

Of the respondents 42% had never used the HMIS data collected at the health facility level for planning, budgeting and evaluation of services provision. This was attributed by almost three quarters (70%) to poor knowledge on data analysis. The other major reasons for failure to utilize the local data were poor quality of data and poor managerial skills reported by 16% and 7% of the respondents respectively.

### Completeness of HMIS booklets

Of all reviewed HMIS booklets only a single delivery register from only one health facility was judged to be 100% complete. These booklets, however, were not filled in as many as 55% of the health facilities (Table 2). The types of information that was found not recorded in the booklets for postnatal services (child vaccination/weight) were measles vaccine, DPT 3, polio vaccine and Vitamin A. These services were not recorded in these booklets despite the fact that it was assumed that these important health interventions had been given to the clients.

**Table 2:** Level of completeness of HMIS booklets from Kilombero district health facilities, 2007

Types of HMIS booklets	Proportions of health facilities, n = 11			
	
	Level of completeness of HMIS booklets			
	100%	75% - 99%	50% - 74%	Never filled the booklets
Postnatal services booklets	0%	73%	0%	27%
Delivery booklets	9%	36%	0%	55%
Antenatal services' booklets	0%	36%	36%	27%

The type of information which was mostly not filled in the delivery booklets was the condition of the mother at discharge. The parameters which were commonly missing in the antenatal services' booklets were pregnant mothers' risk factors, VDRL test, TT vaccination and height. Reasons for such incompleteness found were lack of VDRL reagent, workload pressure, forgetfulness and poor knowledge on data recording.

### Recommendations for HMIS improvement

Almost all respondents (95%) recommended training of health care providers in order to improve HMIS. Almost a quarter (23%) of respondents recommended for improved supervision and increased staffing levels at the facility level. Only 19% recommended for revision and simplification of the HMIS to be more user-friendly. The respondent from the district authority reported that the process of health data in the current HMIS was long and difficult, with many booklets and forms with some repeating information. Poor knowledge on HMIS among health workers was linked to lack of training on the system and workload pressure. In view of these gaps the system was recommended for revision.

## Discussion

Our findings revealed a wide range of interlinked factors responsible for the inefficiency and ineffectiveness of the HMIS in Tanzania. The lack of clear understanding of the purpose, users and flow pattern of health data collection was linked to poor quality of data collection and suggests that decision-making in the country health system may be less than adequately informed. This study has revealed higher proportion (65%) of care providers who failed to define properly what HMIS is than that (47%) reported in previous studies [6]. These findings suggest a declining knowledge on this important system. On the contrary, despite such low knowledge on HMIS, the majority of the care providers (91%) had positive attitude towards the system, indicating substantial acceptability, a positive potential factor for improvement. The existing huge gap of knowledge on such an important system can be linked to lack of training which was as high as 81% of care providers. On the other hand the findings suggest lack of emphasis on HMIS in the pre-service curricula and hence a lack of evidence-based training in medical and paramedical training institutions in the country. Considering the ongoing process to develop and introduce competence-based educational curricula for all medical and paramedical training programs in the country and the government 10 year program to expand training of care providers, incorporation of HMIS in the new curricula is greatly suggested. The government of Tanzania through its 10 year PHSDP (2008 - 2017), intends to train 460,000 health care providers by the year 2017 and improve the provision of health services to the level of every village and ward [10,15]. The author believes that incorporation of HMIS in the new curricula will improve not only knowledge, skills, culture and efficiency of HMIS but will also cut-down the investment required for on-the-job training for health care providers.

The failure to collect health data as seen in 55% of the health facilities for HMIS delivery booklets, indicates the high degree of poor documentation, underreporting and data inaccuracy from the district up to the national level. The HMIS guidelines require health care providers to complete relevant booklets immediately after provision of health care services before the patients or clients leave the facility. The impact of such poor compliance with this system is worrisome and suggests that vital public health decisions are made based on crude district and national estimates of burden of the problems. The failure to use health data collected at the health facility level as reported by 63% of care providers indicates that the primary purpose of data collection is to report to higher levels suggesting a high prevalence of the "*mailbox syndrome*". The "mailbox syndrome" is a phenomenon whereby a crucial information generated at the health facility level is mailed rather than used locally for quality care improvement [16]. This syndrome is contrary to the concept of decentralization which is currently implemented in the country. These findings suggest also that the existing HMIS has not been institutionalized in the sense of being integrated into the everyday activities, an important factor for its sustainability and reliability.

Like many other reports the incompleteness and poor use of health data collected at a health facility, found in this study, can be attributed to poor knowledge on HMIS; inadequate financial, human and technological resource capacity; lack of user-friendly systems; lack of coordination and evaluation, as well as inadequate policies to manage the sustainability of the system [17,18]. Considering the lengthy and laborious HMIS procedural requirements for completion of the booklets and the context of acute shortage of care providers, revision for a more user-friendly system is highly recommended.

The fact that these factors have been documented and remained unattended in Tanzania over many years, indicates a high degree of irresponsiveness and unaccountability in the country health system [19,20]. These findings suggest in part poor leadership performance. Carrying out "*business as usual*", a static mindset among the key actors and poor supervision of health systems are progress blocking agents which have been reported as the leading factors for poor performance of health sectors in sub-Saharan Africa [21]. These findings call for more commitment, dedication and accountability within an HMIS organization [22]. Considering these factors and the fact that Tanzania is already off-track in HMIS, evidence-based John Kotter's eight-step process for implementing successful changes is indicated for effective system in this country. These steps are: to create a sense of urgency for change, create powerful group guiding the change, develop and communicate the change vision and strategy, empower others to act, produce short-term wins, press harder and faster after the first successes and create a new culture for sustainability [11].

## Conclusions

This article has revealed a state of poor health data collection, lack of data-based decision-making at all levels and the factors for change in the country's HMIS. It calls for new innovations including incorporation of HMIS in the ongoing reviews of the educational curricula for all cadres of health care providers, development of more user-friendly system and use of evidence-based John Kotter's eight-step process for implementing successful changes in this system.

## Abbreviations

BCG: Bacille Calmette-Guérin (Tuberculosis) Vaccine; DPT: Diphtheria, Pertussis and Tetanus toxoids; HMIS: Health Management Information System; NACTE: National Council for Technical Education; PHSDP: Primary Health Service Development Program; RCH: Reproductive and child health; VDRL: Venereal Disease Research Laboratory (test for syphilis); TT: Tetanus Toxoid.

## Competing interests

The author declares that they have no competing interests.

## Authors' contributions

ASN designed the study, was involved in data collection and the analyzed the data and wrote the manuscript.

## Pre-publication history

The pre-publication history for this paper can be accessed here:

http://www.biomedcentral.com/1472-6947/10/36/prepub

## Supplementary Material

Additional file 1 **Knowledge, attitude and practice on health management information system in Kilombero district, Tanzania**. A semi-structured questionnaire used to assess training, knowledge, attitude and practice on health management information system among health workers in Kilombero district. The tool also explores the gaps and factors for change in the existing health management information system. Click here for file
