# Supplementary material for: Bridging the gaps in the Health Management Information System in the context of a changing health sector
Source: BMC Med Inform Decis Mak. 2010 Jun 25;10:36. doi: 10.1186/1472-6947-10-36 (PMC2914713; doi:10.1186/1472-6947-10-36)
Supplement: Additional file 1 — Knowledge, attitude and practice on health management information system in Kilombero district, Tanzania. A semi-structured questionnaire used to assess training, knowledge, attitude and practice on health management information system among health workers in Kilombero district. The tool also explores the gaps and factors for change in the existing health management information system. [file 1472-6947-10-36-S1.DOC]

**KNOWLEDGE, ATTITUDE AND PRACTICE ON HEALTH MANAGEMENT INFORMATION SYSTEM IN KILOMBERO DISTRICT, TANZANIA**

**Introduction**

This study is being conducted to assess the implementation of HMIS in Tanzania, and it specifically seeks to understand the factors that may affect its effective implementation at the health facility level. In this interview I am specifically interested in getting your inputs and experiences, the problems you face or you are aware of, success stories and recommendations you have. I will then compile a report that will contain all staff comments without any reference to individuals. Your comments will remain confidential and will not be accessed by any other person except the researcher.

**If you agree, I will then proceed with the interview.**

**Instruction to interviewer**

**DO NOT READ the coding options for the interviewee. Probe for responses as many as possible.**

| **SN** |  | | | **CODING CATEGORIES** | | **CODE** |
| --- | --- | --- | --- | --- | --- | --- |
| **Part I: Health Facility Information** | | | | | | |
| 1.1 | Name of Facility | | | **____________________________** | |  |
| 1.2 | Category of facility | | | 1. Hospital  2. Health centre  3. Dispensary | | /___/ |
| 1.3 | Facility ownership | | | 1. Government  2. Religious  3. Private | | /___/ |
| **Part II: Interviewee’s Background Information** | | | | | | |
| 2.1 | Responsibility at the facility level | | | 1. Facility in charge  2. Head of unit  3. Health care provider | | /___/ |
| 2.2 | Designation | | | 1. Medical Attendant 2. Enrolled Nurse 3. Nurse Officer 4. Assistant Clinical Officer 5. Clinical Officer 6. Medical Officer/Assistant | | /___/ |
| 2.3 | Working experience | | | 1.Less than five years  2.Five years and above | | /___/ |
| 2.4 | Ever exposed to any formal training on HMIS? | | | 1.Yes  2.NO (if **NO** go to part iii) | | /___/ |
| 2.5 | If Yes in Question 2.4, when did you have your last training done? | | | 1.Less than 5 years  2.Five or more years ago | | /___/ |
| **Part III: Knowledge on HMIS** | | | | | | |
| 3.1 | How would you define the term Health Management Information System (HMIS)? | | | _________________________________________  _________________________________________  _________________________________________ | | |
| 3.2 | Can you mention the booklets which are used in HMIS? | | | 1.Yes  2.No | /___/ | |
| 3.3 | If the answer is “YES” in question number 3.2 above mention them  *[Use the checklist of the booklets]* | | | 1. Mentioned all 2. Mentioned 7-9 3. Mentioned 5-6 4. Mentioned 1–5 5. Failed to mention even one | **/___/** | |
| 3.4 | What is the importance of HMIS? | | | 1. ______________________________________ 2. ______________________________________ 3. ______________________________________ | | |
| 3.5 | Where is the HMIS information (data) supposed to be sent?  *Judge whether he/she outlined the information flow pattern correctly, partially, completely incorrectly/does know* | | | 1. Correct  2. Partially correct  3. completely incorrect/does know | /___/ | |
| 3.6 | Who is supposed to utilize the information which you always collect and record in the HMIS registers/booklets? | | | 1.Health manager at facility level  2.Facilities health committee  3.Health Managers at district level  4.Health managers at national level  5.Others (specify)  _____________________________ | *Circle all responses* | |
| 3.7 | How frequently is the HMIS information/ report supposed to be sent to higher levels? | | | 1. Once yearly  2. Twice yearly  3. Quarterly  4. Other frequencies: specify _______ | **/___/** | |
| **Part IV: Attitude of the interviewee towards HMIS** | | | | | | |
| 4.1 | Considering that health care providers have different feelings about HMIS, do you really feel that HMIS is worthy for the time and other resources spent filling and processing data for? | | | 1.Yes  2.No | **/___/** | |
| 4.2 | If Yes/No in question 4.1 above give reasons | | | 1. ___________________________________ 2. ___________________________________ 3. ___________________________________ | | |
|  | **A lot has been said about HMIS by the health care providers, please indicate whether you agree or disagree with the following statements**  **(*Read out the statements)*** | | | |  | |
| 4.4 | It is important to continue with HMIS and associated activities for health care improvement at the health facility level | | 1.Yes  2. No  3. I Don’t know | | **/___/** | |
| 4.5 | HMIS is meant for health managers at the district and ministerial levels and not the health facility. | | 1.Yes  2. No  3. I Don’t know | | **/___/** | |
| 4.6 | The current HMIS is difficult, complicated and need to be simplified. | | 1.Yes  2. No  3. I Don’t know | | /___/ | |
| **Part V: Practice on HMIS** | | | | | | |
| 5.1 | Do you collect and record data in the HMIS booklets when attending patients/ clients? | 1. Yes  2. No | | | /___/ | |
| 5.2 | Who are the consumers of the data which are always collected and recorded HMIS booklets in this facility? | 1. Health manager at facility level  2. Facilities health committee  3. Health Managers at district level  4. Health managers at national level  5. Others (specify)_________________ | | | *Circle all responses* | |
| 5.3 | For what purposes do these consumers utilize the data? | 1. Policy-making 2. Planning and budgeting 3. Evaluation of health programs 4. Others (specify _____________________________ | | | /___/ | |
| 5.4 | From your experience how do you utilize these data in this facility?  (*Probe the interviewee to give few examples on how they utilized the HMIS data for health care improvement)* | 1. ______________________________________________________________________________ 2. ______________________________________________________________________________ 3. **______________________________________________________________________** | | | | |
| 5.5 | What are the factors, if any that hinder you not to effectively utilize the information recorded in the HMIS register? | 1. The current structure of the health  system does not encourage local  utilization of the data  2. Poor knowledge on the data analysis  3. Poor managerial skills  4. Poor quality of data  5. Other reasons (specify)  _______________________________  _______________________________ | | | *Circle all responses* | |
| 5.6 | In your opinion (if any) what should be done in order to improve the HMIS? | 1. _______________________________________ 2. _______________________________________ 3. _______________________________________ 4. _______________________________________ | | | | |
